# Supplementary material for: Inhibitory Activity of Hydroxypropyl Methylcellulose on Rhinovirus and Influenza A Virus Infection of Human Nasal Epithelial Cells
Source: Viruses. 2025 Mar 6;17(3):376. doi: 10.3390/v17030376 (PMC11946253; doi:10.3390/v17030376)
Supplement: Supplementary file 1 [file viruses-17-00376-s001.zip › viruses-3514340-supplementary.pdf]

Supplementary Materials and Methods

Derivation of hNESPCs and In Vitro Differentiation of hNECs

The hNECs were differentiated from human nasal epithelial stem/progenitor cells (hNESPCs) as described previously [26,27]. Nasal tissue biopsies were dissociated into single cells, and were seeded onto NIH/3T3 feeder layer cells (ATCC) treated with mitomycin-C (Sigma). The hNESPCs were grown to confluency using medium 3 (serum-free), and then seeded onto 12-mm diameter Transwell inserts with 0.4-μM polyester membrane (Corning) for air-liquid interface (ALI) culture. The PneumaCult-ALI medium (STEMCELL Technologies) was used as differentiation medium, and was refreshed every 48 h. The hNECs were incubated for 10 min in 1× dPBS which was then discarded to remove secreted mucus in the apical chamber during change of medium. Fully differentiated hNECs, with beating ciliated cells and mucus-producing goblet cells, were obtained after 21 to 28 days of ALI culture.

Virus Infection of Fully Differentiated hNECs

The hNECs were washed once using 1× dPBS for 10 min at 37 °C. 100 μL each of HRV16 (MOI of 2.5), H1N1 (MOI of 0.01) and H3N2 (MOI of 0.01) were used for infection. The multiplicity of infection (MOI) was calculated based on the total number of cells in each Transwell. The hNECs were then incubated for 1 h at 33 °C (HRV) or 35 °C (H1N1 and H3N2) before the virus inoculum was removed. 100 μL of 1× dPBS was incubated in the apical chamber of infected and mock-infected hNECs for 10 min at 33 °C (HRV) or 35 °C (H1N1 and H3N2) to collect the supernatant as the apical secretion. Apical supernatant of virus-infected and mock-infected hNECs were collected at 24 hpi for the relevant assays.

Viral Titer Quantification using Plaque Assay

Viral titer quantification using the plaque assay was performed as described previously [26,27]. HeLa cells or MDCK cells at 85 to 95% confluency in 24-well plates were incubated with 100 μL of serial-diluted RV or IAV supernatant samples from infected hNECs at 33 °C or 35 °C for 1 h respectively. The inoculum was removed, replaced with 1 mL of Avicel (FMC Biopolymer) overlay to each well, and incubated at 33 °C or 35 °C for 72 h. Avicel overlay was removed after the incubation period, cells were fixed with 4% formaldehyde in 1× PBS for 1 h, and stained with 1% crystal violet. PFU values were calculated as follows:

Number of plaques × Dilution factor = Number of PFU per 100 μL.

Cell viability assay

Cell viability was assessed using the AlamarBlue cell viability assay (Thermo Fisher). The hNECs were dissociated by trypsinization, and 10% AlamarBlue was added to each sample. 100 uL of each sample was added into each well in triplicate, and plates were incubated at 37 °C for 2 h. Absorbance was measured at 570 nm and 600 nm using a BioTek Synergy

plate reader, and the percentage reduction was calculated according to the following equation:

$$\text{Percent reduced} = \frac{(\varepsilon_{OX})\lambda_2 A\lambda_1 - (\varepsilon_{OX})\lambda_1 A\lambda_2}{(\varepsilon_{RED})\lambda_1 A'\lambda_2 - (\varepsilon_{RED})\lambda_2 A'\lambda_1} \times 100$$

Cell viability was assessed using the CyQUANT LDH cytotoxicity assay (Thermo Fisher). Briefly, the hNECs on the transwell membrane were washed in 100 µL of 1× dPBS at 37 °C for 10 min and collected. Next, 50 µL of each sample was added into each well in duplicate, and the plates were incubated at room temperature for 30 min. Absorbance was measured at 490 nm and 680 nm, and the percentage reduction was expressed as a percentage of total LDH released upon lysis of the hNECs using the lysis buffer provided in the kit.

Measurement of Ciliary Beating Frequency of hNECs

Ciliary beating frequency (CBF) was auto-analyzed using the Sisson-Ammons video analysis (SAVA) system (Ammons Engineering, Clio, MI, USA) as previously described [26]. Whole field analysis was performed by using software that automatically analyzed the entire captured video of all ciliated cells in a given field. For all experiments, the digital sampling rate was set at 100 frames per second (fps). The predominant frequency of a small group of cilia from each sample was viewed and recorded at a minimum of three separate fields every 1 min for up to 3 min while they were maintained at a constant temperature (23 ± 0.5 °C).

Immunofluorescence Staining

For mock- and RV-infected hNECs, the fixed cells on cytospin slides were permeabilized with 0.1% Triton X-100 for 10 min, washed and blocked with 10% goat serum (Invitrogen) before incubation with primary antibodies overnight. To stain for IAV NS1, the fixed mock- and H1N1-infected hNECs on cytospin slides were subjected to antigen retrieval in Tris-EDTA buffer pH 9 (Abcam) and heating for 10 to 15 min at 95 °C. After washing briefly in 1× PBS, the cells were permeabilized with 0.25% Triton X-100 for 10 min, washed and blocked with 10% goat serum (Invitrogen) before incubation with primary antibodies overnight. Rabbit and mouse monoclonal antibody against acetylated α-tubulin (1:5000, ab179484, ab24610; Abcam), mouse monoclonal antibody against HRV VP2 (1:1000, #18758; QED Biosciences) and rabbit polyclonal antibody against H1N1 NS1 (1:200, #PA5-32243; Invitrogen) were used for immunofluorescence (IF) staining. Alexa Fluor 488 (anti-rabbit a11034, anti-mouse a11029) and 594 (anti-rabbit a11037, anti-mouse a11032) were used at dilution of 1:500 (Life Technologies), and ProLong AntiFade mounting medium with DAPI was used to mount the slides (Life Technologies). The IF images were captured using a fluorescence microscope (Olympus IX51).

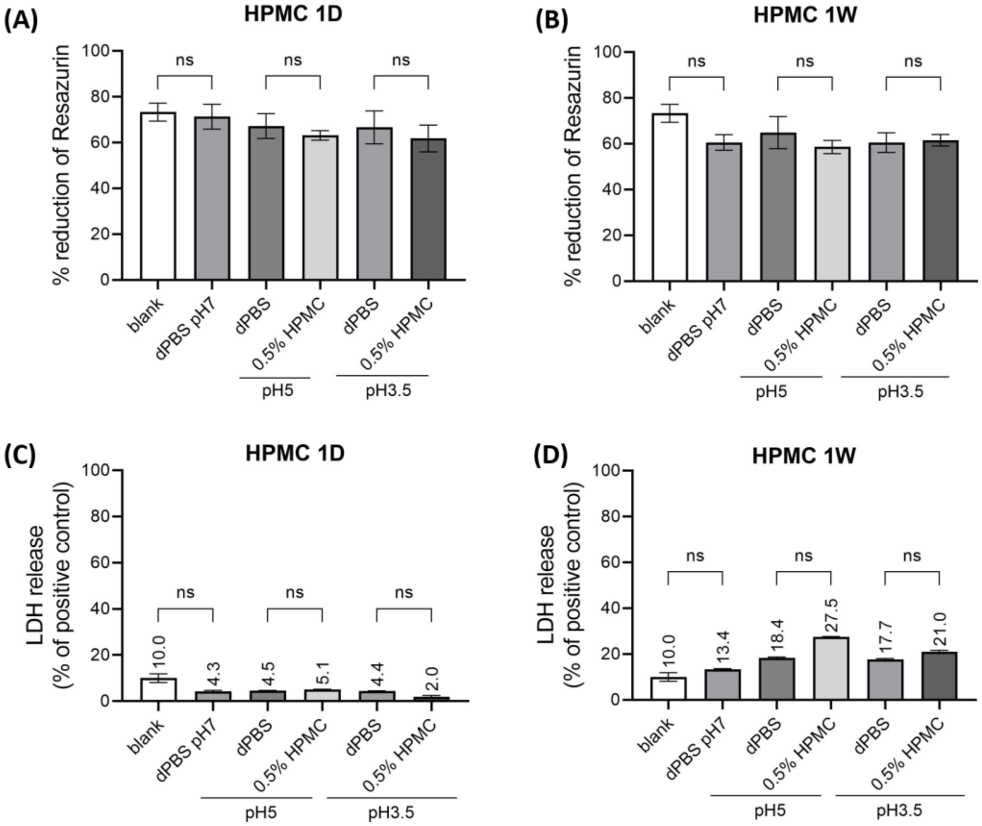

Supplementary Figure S1. One-day and one-week HPMC treatment in acidic buffer (pH 3.5 and pH 5) do not induce significant cell death of hNECs. (A,B) There was no significant change in the percentage reduction of resazurin by total live cells, and hence no significant alteration in cellular metabolic activity was detected for hNECs treated with 0.5% HPMC at pH 3.5 and pH 5, and acidic buffer alone (1× dPBS at pH 3.5 and pH 5). Percentage reduction of resazurin of untreated hNECs (blank) and hNECs with 1× dPBS (pH 7) were also measured for comparison. (C,D) Cell viability assessed by LDH assay using apical supernatant of hNECs showed that there was no significant change in the LDH released by dead cells for hNECs treated with 0.5% HPMC at pH 3.5 and pH 5, and acidic buffer alone (1× dPBS at pH 3.5 and pH 5).

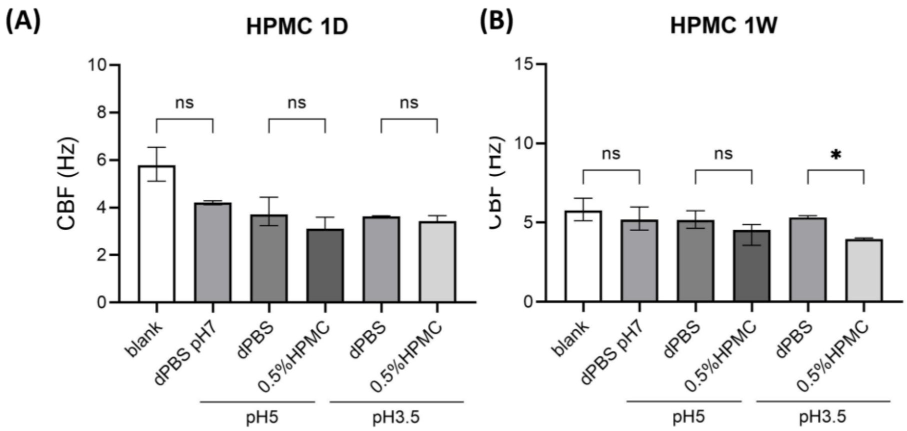

Supplementary Figure S2. Effects of one-day and one-week 0.5% HPMC treatment in different acidic buffers (pH 3.5 and pH 5) on CBF of hNECs. (A) One-day (1D) HPMC treatment in acidic buffer (pH 3.5 and pH 5), and (B) one-week (1W) treatment of HPMC at pH 5 did not induce significant reduction in CBF of hNECs. However, the CBF of hNECs was slightly reduced following one-week treatment of 0.5% HPMC at pH 3.5.

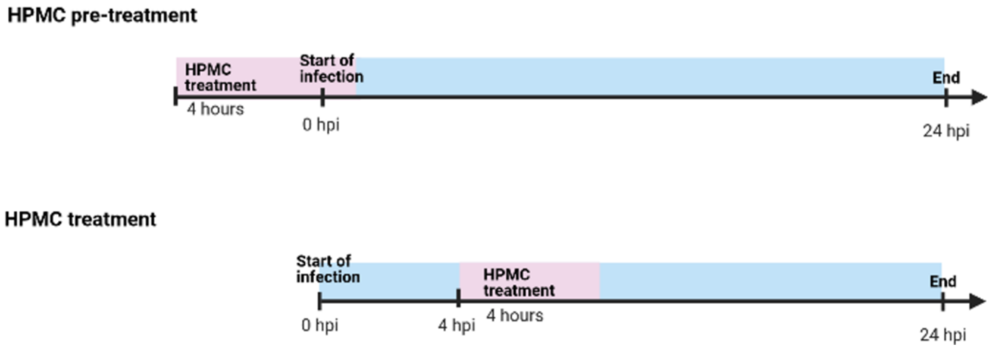

Supplementary Figure S3. Timeline of HPMC treatment before and after virus infection of hNECs. For pre-treatment experiments, HPMC was added onto hNECs 4 h prior to start of infection. Virus inoculum was added directly onto HPMC for 1 h before removal of all solutions. For post-infection treatment experiments, HPMC was added onto hNECs at 4 hpi (for 4 hours).

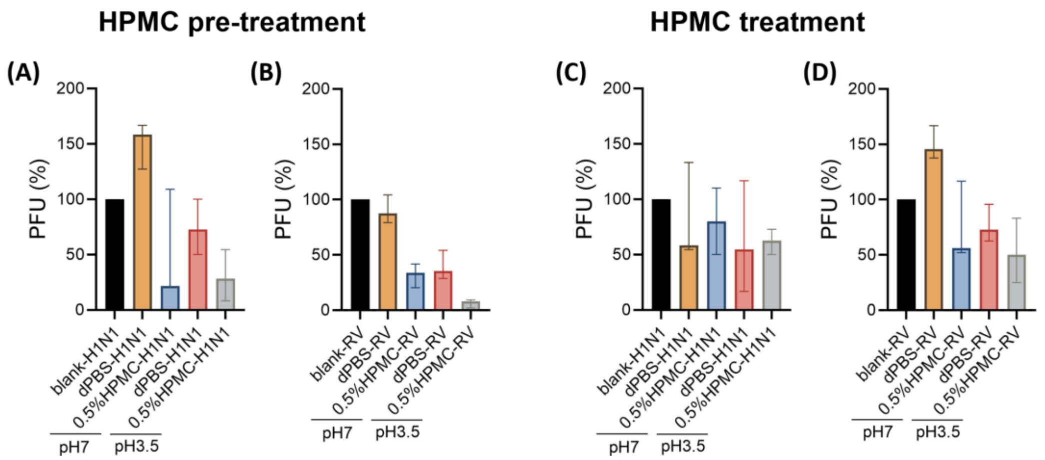

Supplementary Figure S4. Additive effect of treatment using HPMC and acidic buffer before and after infection in reducing viral progeny production in hNECs. (A,B) The pre-infection treatment of hNECs using acidic buffer alone reduced the release of H1N1 and RV progeny production, and the viral titers were further decreased by pre-treatment using 0.5% HPMC in acidic buffer. H1N1 and RV titers were also reduced when hNECs were pre-treated using 0.5% HPMC in pH-neutral buffer. However, pre-treatment of hNECs using only pH-neutral buffer before IAV infection resulted in increased viral titer (*n* = 3). (C,D) Post-infection treatment of hNECs using acidic buffer alone or using 0.5% HPMC in acidic or pH-neutral buffer could reduce the release of IAV and RV progeny production. However, post-infection treatment of RV-infected hNECs using only pH-neutral buffer culminated in an increase in viral titer.

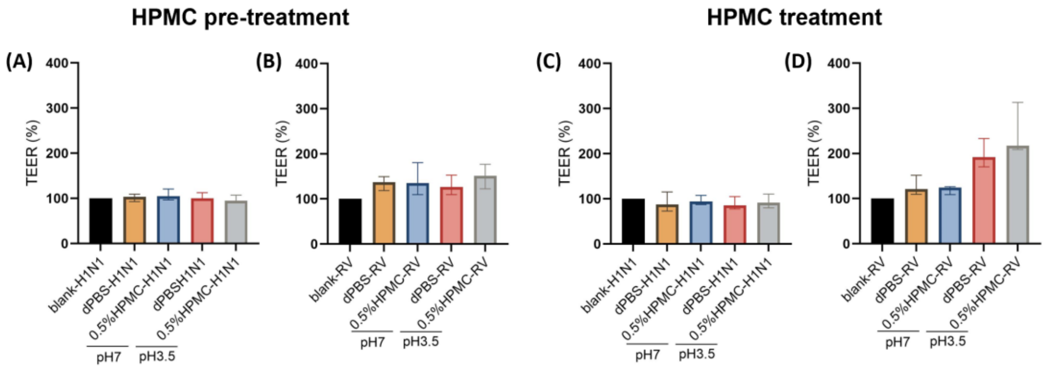

Supplementary Figure S5. No significant change in trans-epithelial electrical resistance (TEER) of hNECs for treatment with PBS and HPMC at pH 7 and pH 3.5 before and after infection with H1N1 and RV (at 24 hpi). (A,B) The pre-infection treatment of hNECs using acidic buffer or neutral buffer, and 0.5% HPMC in acidic buffer and neutral buffer did not significantly change TEER as compared to the untreated control (blank) (*n* = 3). (C,D) The post-infection treatment of hNECs using acidic buffer or neutral buffer, and 0.5% HPMC in acidic buffer and neutral buffer did not significantly change TEER as compared to the untreated control (blank) (*n* = 3), although increased TEER was observed for treatment at pH 3.5 for RV infection.

Supplementary Table S1. Patient information of donors.

Code	Ethnicity	Age	Gender	Allergy*	Asthma#	Smoker
J1	Chinese	50	F	No	No	No
J2	Chinese	56	M	No	No	No
J3	Chinese	21	M	No	No	No
J4	Chinese	35	M	No	No	No
J5	Others	32	M	No	No	No
J6	Chinese	51	F	No	No	No
J7	Chinese	34	F	No	No	No
J8	Indian	41	M	No	No	No
J9	Chinese	44	F	No	No	No
J10	Others	27	M	No	No	No
J11	Chinese	31	M	No	No	No
J12	Others	54	M	No	No	No

F: female; M: male.

* Diagnosis of allergic rhinitis was based on the concordance between a typical history of allergic symptoms and skin prick testing using a local panel of common allergens.

# Diagnosis or history of asthma was based on medical records kept at the National University Hospital, Singapore.

Supplementary Table S2. **Virus levels of hNECs from 3 donors (PFU/mL) for treatment using pH 3.5 and pH 7 buffer alone and using HPMC in acidic and neutral buffer before and after H1N1 and RV infection.**

		HPMC Treatment Before Infection				
		**Blank**	**PBS pH 7**	**HPMC pH 7**	**PBS pH 3.5**	**HPMC pH 3.5**
**H1N1**	Donor 1	60,000	95,000	13,000	30,000	5000
	Donor 2	30,000	50,000	6000	30,000	8500
	Donor 3	5500	7000	6000	4000	3000
**RV**	Donor 1	400,000	350,000	135,000	115,000	8,000
	Donor 2	120,000	95,000	24,500	65,000	10,000
	Donor 3	240,000	250,000	100,000	85,000	22,500

		HPMC Treatment After Infection				
		**Blank**	**PBS pH 7**	**HPMC pH 7**	**PBS pH 3.5**	**HPMC pH 3.5**
**H1N1**	Donor 1	60,000	35,000	30,000	10,000	80,000
	Donor 2	30,000	40,000	45,000	1000	40,000
	Donor 3	5500	3000	8000	7000	10,000
**RV**	Donor 1	400,000	550,000	225,000	250,000	100,000
	Donor 2	120,000	250,000	140,000	115,000	100,000
	Donor 3	240,000	750,000	125,000	175,000	120,000

Supplementary Table S3. **The median values of TEER (ohm/cm^2) for treatment using pH 3.5 and pH 7 buffer alone and using HPMC in acidic and neutral buffer before and after H1N1 and RV infection of hNECs. The mock control indicates uninfected hNECs.**

		**Mock**	**H1N1**	**RV**
**Blank control**		1298	2050	910
**HPMC treatment before infection**	PBS pH 3.5	1255	1994	909
	HPMC pH 3.5	1243	1912	730
	PBS pH 7	1459	2120	985
	HPMC pH 7	1492	2146	1018
**HPMC treatment after infection**	PBS pH 3.5	1722	1764	1404
	HPMC pH 3.5	1892	1858	1527
	PBS pH 7	1943	1786	884
	HPMC pH 7	1832	1922	923

Supplementary Table S4. Virus levels of hNECs from 6 donors (PFU/mL) for HPMC treatment in pH 3.5 buffer before and after infection with H3N2, H1N1 and RV.

**HPMC Treatment Before Infection**				
		**Blank**	**PBS pH 3.5**	**HPMC pH 3.5**
**H3N2**	Donor 1	320,000	240,000	200,000
	Donor 2	225,000	150,000	115,000
	Donor 3	210,000	150,000	900
	Donor 4	250,000	28,000	20,000
	Donor 5	120,000	80,000	20,000
	Donor 6	17,000	8500	6000
**H1N1**	Donor 1	300,000	260,000	150,000
	Donor 2	200,000	180,000	210,000
	Donor 3	180,000	15,000	10,000
	Donor 4	210,000	14,000	10,000
	Donor 5	350,000	300,000	125,000
	Donor 6	15,000	8000	4500
**RV**	Donor 1	185,000	100,000	70,000
	Donor 2	335,000	66,000	17,500
	Donor 3	600,000	300,000	210,000
	Donor 4	245,000	13,500	55,000
	Donor 5	450,000	120,000	45,000
	Donor 6	1,100,000	150,000	300,000
**HPMC Treatment After Infection**				
		**Blank**	**PBS pH 3.5**	**HPMC pH 3.5**
**H3N2**	Donor 1	320,000	110,000	115,000
	Donor 2	225,000	28,000	150,000
	Donor 3	210,000	10,000	12,000
	Donor 4	250,000	10,000	22,000
	Donor 5	120,000	2800	9000
	Donor 6	17,000	7500	1550
**H1N1**	Donor 1	300,000	250,000	150,000
	Donor 2	200,000	32,000	21,000
	Donor 3	180,000	6000	18,000
	Donor 4	210,000	15,000	25,000
	Donor 5	350,000	700	115,000
	Donor 6	15,000	1050	12,500
**RV**	Donor 1	185,000	80,000	33,000
	Donor 2	335,000	350,000	110,000
	Donor 3	600,000	400,000	100,000
	Donor 4	245,000	195,000	110,000
	Donor 5	450,000	300,000	300,000
	Donor 6	1,100,000	950,000	300,000

Supplementary Table S5. The pH strip test of acidic buffer alone and HPMC in acidic buffer before administration onto hNECs, and after 4 hours treatment on hNECs.

Sample	pH Strip Test	pH Reading
PBS at pH 3.5 before adding to hNECs	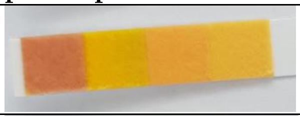	~ 3
HPMC in PBS at pH 3.5 before adding to hNECs	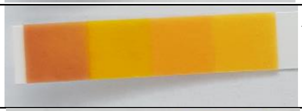	~ 3
PBS at pH 3.5 removed after 4 h of treatment on hNECs	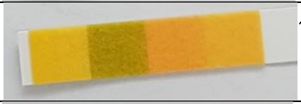	~ 5
HPMC at pH 3.5 removed after 4 h of treatment on hNECs	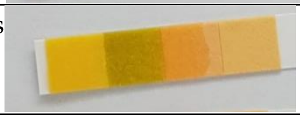	~ 5

References

26. Ong, H.H.; Andiappan, A.K.; Duan, K.; Lum, J.; Liu, J.; Tan, K.S.; Howland, S.; Lee, B.; Ong, Y.K.; Thong, M.; et al. Transcriptomics of rhinovirus persistence reveals sustained expression of RIG-I and interferon-stimulated genes in nasal epithelial cells in vitro. Allergy 2022, 77, 2778–2793. https://doi.org/10.1111/all.15280.

27. Ong, H.H.; Liu, J.; Oo, Y.; Thong, M.; Wang, D.Y.; Chow, V.T. Prolonged Primary Rhinovirus Infection of Human Nasal Epithelial Cells Diminishes the Viral Load of Secondary Influenza H3N2 Infection via the Antiviral State Mediated by RIG-I and Interferon-Stimulated Genes. Cells 2023, 12, 1152. https://doi.org/10.3390/cells12081152.
